# Emergence of aspirin as a promising chemopreventive and chemotherapeutic agent for liver cancer

**DOI:** 10.1038/cddis.2017.513

**Published:** 2017-10-12

**Authors:** Hongping Xia, Kam M Hui

**Affiliations:** 1 Laboratory of Cancer Genomics, Division of Cellular and Molecular Research, National Cancer Centre, Singapore, Singapore; 2 Department of Pathology, School of Basic Medical Sciences & The Affiliated Sir Run Run Hospital, Nanjing Medical University, Nanjing, China; 3 Cancer and Stem Cell Biology Program, Duke-NUS Medical School, Singapore, Singapore; 4 Department of General Surgery, Singapore General Hospital, Singapore, Singapore; 5 Institute of Molecular and Cell Biology, A*STAR, Biopolis Drive Proteos, Singapore, Singapore

Aspirin is a non-steroidal anti-inflammatory drug (NSAID) that has been established to treat pain, fever and inflammation. Evidence demonstrated that whenever a cell is injured, prostaglandin is released and NSAIDs such as aspirin can help prevent and relieve these symptoms of injury by blocking the action of cyclooxygenase (COX), the enzyme responsible for the synthesis of prostaglandins. Aspirin acts by acetylating platelet COX-1 and as a consequence, also irreversibly inhibiting platelet function.^1^ Indeed, by exerting its antiplatelet effects, aspirin could significantly reduce the risk of myocardial infarction and stroke. Substantial evidence have also demonstrated the potential of aspirin for the prevention and treatment of cancer. Multiple clinical studies have demonstrated a link between long-term aspirin use and a reduction in the incidence and mortality of several cancer types, including colorectal, stomach, esophageal, breast, lung, prostate and liver cancers.^2, 3^ In this News and View, we have highlighted the emergence of aspirin as a chemoprevention agent and its role as an adjuvant therapy in cancer, focusing mainly on hepatocellular carcinoma (HCC) (Figure 1).

Because of the dismal prognosis of HCC, chemoprevention offers an appealing strategy. In 2012, it was demonstrated in the chronic hepatitis B (CHB) mouse model that dual antiplatelet therapy with aspirin and clopidogrel could prevent HCC and improve survival.^4^ HBV transgenic mice treated with dual aspirin–clopidogrel therapy showed reduction in overall liver damage, inflammation and fibrosis. The dual therapy also reduced disease progression and prolonged overall survival.^4^ By analyzing data from the Taiwan National Health Insurance Research Database, the use of aspirin or clopidogrel was also significantly associated with better overall survival and disease-free survival for patients with hepatitis B virus (HBV)-related HCC following liver resection surgery.^5^ To explore the protective effects of antiplatelet therapy against HCC, Lee *et al.* conducted a retrospective analysis of the risk of HCC in 1674 patients with CHB and whose HBV DNA levels were suppressed by antivirals to <2000 IU/ml. Risk was compared between patients received antiplatelet treatment (aspirin, clopidogrel or both) and patients who were not treated. The primary and secondary outcomes were development of HCC and bleeding events, respectively. During the study period, the antiplatelet-treated group showed a significantly lower risk of HCC compared to the untreated, regardless of the antiplatelet agent. While treatment with aspirin alone was not associated with a higher bleeding risk, however, antiplatelet therapy containing clopidogrel may increase the overall risk of bleeding.^3^ A nationwide cohort study from Taiwan has also concurred that NSAIDs or aspirin use associated with a reduced risk of HCC recurrence.^6^ Moreover, an independent study from The National Institutes of Health (NIH) of the United States has also shown that aspirin use was associated with a decreased risk of developing HCC and death from chronic liver disease (CLD), whereas nonaspirin NSAID use was only associated with reduced risk of CLD death.^7^

Besides evidence emerging to support aspirin has potential value of chemoprevention, the antitumor effects of aspirin have also been examined. The association between platelets and cancer progression is well recognized and multiple mechanisms have been proposed to explain the complex interactions between platelets and tumor cells. Aspirin is an antiplatelet drug and its anticancer activity has been extensively investigated using cancer cell lines, animal models as well as clinical trials.^8^ Randomized controlled trials also showed that aspirin reduced the risk of metastasis of adenocarcinomas, especially in patients with metastatic colorectal cancer.^9^

Sorafenib is currently the only FDA-approved molecular inhibitor for the systemic therapy of advanced HCC. However, its high cost, a marginal benefit increase and often severe side effects are the major clinical challenges associated with the treatment of HCC with sorafenib. Being an example of inflammation-related cancer and the chronic inflammatory state in response to liver damage and viral infection appears to be necessary for its initiation and development, the syngergistic therapeutic effect with dual treatment of sorafenib and aspirin has therefore been explored. Aspirin in combination with TACE has been reported to improve overall survival in treating patients with unresectable HCC.^10^ Moreover, significant survival improvement was also reported when aspirin was taken at the time of embolization for HCC.^11^ Also, there is a current prospective randomized controlled trial registered in China to investigate the effect of sorafenib combined with aspirin in preventing patient risk for postoperative surgical recurrence of HCC (NCT02748304). The primary outcome of the trial is to measure the 5-year overall survival and the secondary outcome is to measure the 5-year disease-free survival and treatment-related bleedings. Unfortunately, the design of most of these trials suffer from the lack of biomarker-targeted selection of subpopulations of HCC patients.

As appropriate patient selection has been implicated to contribute significantly for achieving clinically meaningful results in cancer drug discovery and development, our recent publication addresses the mechanistic aspects of the synergistic effects of aspirin and sorafenib in the combination therapy for HCC.^12^ Previous studies have suggested that the observed pro-metastasis effect of sorafenib resulted from the downregulation of the expression of oxidoreductase HTATIP2, a tumor suppressor in HCC and stromal cell-derived factor 1-*α* (SDF1-*α*) expression in tumor microenvironment of HCC. Aspirin suppresses COX2 and SDF1-*α* expression leading to the increase expression of HTATIP2 and therefore counteracts the pro-invasion and pro-metastasis effects of sorafenib in HCC.^13, 14^ However, we observed that the expression of HTATIP2 was not significantly modulated when expression data were analyzed in the TCGA-LIHC public data set (http://cancergenome.nih.gov/) and in a data set established in our laboratory for HBV-related HCC (access at Array Express with the codes E-MEXP-84 and E-TABM-292).^15^ Therefore, the synergistic antitumor effect observed for aspirin and sorafenib cannot be attributed solely to the upregulation of HTATIP2. In our recent publication,^12^ we demonstrated that the synergism observed with combining sorafenib and aspirin was mediated by the repression and activation of specific apoptosis-related genes. We have demonstrated that by combining low-dose sorafenib and aspirin, the synergistic antitumor effects observed are related to the simultaneously silencing of long-chain-fatty-acid CoA ligase 4 (ACSL4) and the induction of growth arrest and DNA damage inducible beta (GADD45B) expression. Furthermore, clinical evidence has independently corroborated that survival of HCC patients expressing ACSL4^high^GADD45B^low^ was significantly poorer compared to patients with ACSL4^low^GADD45B^high^ expression, thus demonstrating the potential clinical value of combining aspirin and sorafenib to treat HCC patients expressing ACSL4^high^GADD45B^low^, offering a clear strategy of evidence-driven precision medicine in HCC. In conclusion, aspirin has emerged as a promising chemopreventive and chemotherapeutic agent for HCC.

## Publisher’s Note

Springer Nature remains neutral with regard to jurisdictional claims in published maps and institutional affiliations.

## Figures and Tables

**Figure 1:**
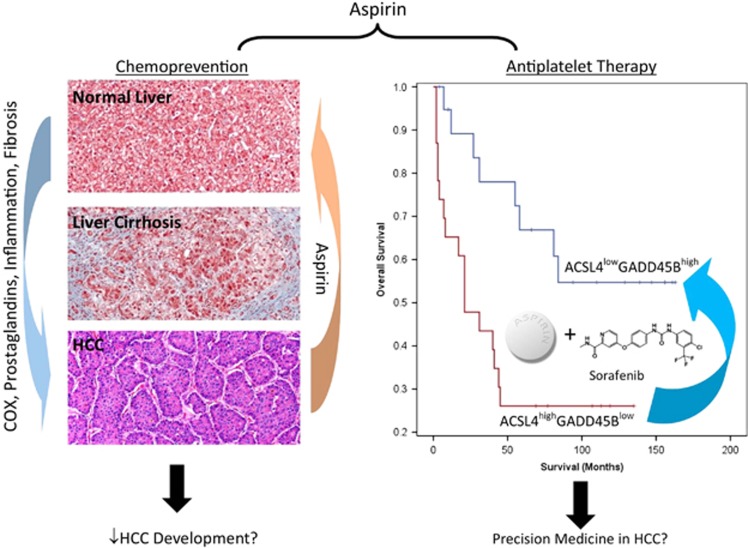
The dual roles of aspirin in the chemoprevention and adjuvant therapy for HCC. Emerging evidence support the potential chemopreventive value of aspirin in protecting the liver against fibrosis through suppression of inflammation. Moreover, recent evidence also show that survival of HCC patients expressing ACSL4^high^GADD45B^low^ was significantly poorer compared to patients with ACSL4^low^GADD45B^high^ expression. The observation that aspirin and sorafenib suppress HCC through the downregulation of ACSL4 and upregulation of GADD45B suggests the potential clinical value of combining aspirin and sorafenib for the target treatment of HCC patients expressing ACSL4^high^GADD45B^low^, offering a clear strategy of precision medicine in HCC
